# Anti-cancer potential of synergistic phytochemical combinations is influenced by the genetic profile of prostate cancer cell lines

**DOI:** 10.3389/fnut.2023.1119274

**Published:** 2023-03-07

**Authors:** Carol A. Gano, Shadma Fatima, Timothy W. Failes, Gregory M. Arndt, Mila Sajinovic, David Mahns, Ahmad Saedisomeolia, Jens R. Coorssen, Joseph Bucci, Paul de Souza, Fatemeh Vafaee, Kieran F. Scott

**Affiliations:** ^1^School of Medicine, Western Sydney University, Campbelltown, NSW, Australia; ^2^Ingham Institute of Applied Medical Research, Liverpool, NSW, Australia; ^3^School of Biotechnology and Biological Sciences, UNSW Sydney, Sydney, NSW, Australia; ^4^ACRF Drug Discovery Centre, Children’s Cancer Institute, Lowy Cancer Research Centre, UNSW Sydney, Sydney, NSW, Australia; ^5^School of Clinical Medicine, UNSW Medicine and Health, UNSW Sydney, Sydney, NSW, Australia; ^6^School of Human Nutrition, McGill University, Sainte Anne-de-Bellevue, QC, Canada; ^7^Departments of Health Sciences and Biological Sciences, Faculties of Applied Health Science, and Mathematics and Science, Brock University, St. Catharines, ON, Canada; ^8^St George Hospital Clinical School, UNSW, Kogarah, NSW, Australia; ^9^UNSW Data Science Hub (uDASH), UNSW Sydney, Sydney, NSW, Australia

**Keywords:** phytochemicals, pTEN loss, prostate cancer, Bliss, synergistic combinations, network pharmacology

## Abstract

**Introduction:**

Despite strong epidemiological evidence that dietary factors modulate cancer risk, cancer control through dietary intervention has been a largely intractable goal for over sixty years. The effect of tumour genotype on synergy is largely unexplored.

**Methods:**

The effect of seven dietary phytochemicals, quercetin (0–100 μM), curcumin (0–80 μM), genistein, indole-3-carbinol (I3C), equol, resveratrol and epigallocatechin gallate (EGCG) (each 0–200 μM), alone and in all paired combinations om cell viability of the androgen-responsive, pTEN-null (LNCaP), androgen-independent, pTEN-null (PC-3) or androgen-independent, pTEN-positive (DU145) prostate cancer (PCa) cell lines was determined using a high throughput alamarBlue^®^ assay. Synergy, additivity and antagonism were modelled using Bliss additivism and highest single agent equations. Patterns of maximum synergy were identified by polygonogram analysis. Network pharmacology approaches were used to identify interactions with known PCa protein targets.

**Results:**

Synergy was observed with all combinations. In LNCaP and PC-3 cells, I3C mediated maximum synergy with five phytochemicals, while genistein was maximally synergistic with EGCG. In contrast, DU145 cells showed resveratrol-mediated maximum synergy with equol, EGCG and genistein, with I3C mediating maximum synergy with only quercetin and curcumin. Knockdown of pTEN expression in DU145 cells abrogated the synergistic effect of resveratrol without affecting the synergy profile of I3C and quercetin.

**Discussion:**

Our study identifies patterns of synergy that are dependent on tumour cell genotype and are independent of androgen signaling but are dependent on pTEN. Despite evident cell-type specificity in both maximally-synergistic combinations and the pathways that phytochemicals modulate, these combinations interact with similar prostate cancer protein targets. Here, we identify an approach that, when coupled with advanced data analysis methods, may suggest optimal dietary phytochemical combinations for individual consumption based on tumour molecular profile.

**Graphical abstract:**
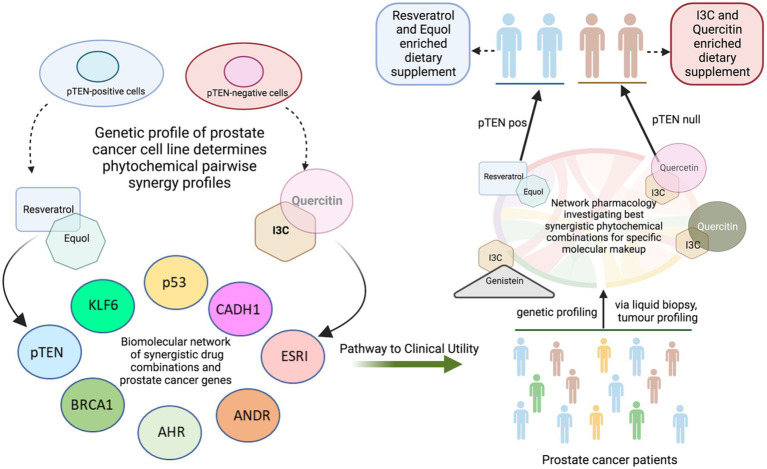

## 1. Introduction

Traditional regional cuisines of Asia and the Mediterranean contain unique phytochemicals that epidemiological studies indicate are of benefit in the prevention of prostate cancer (PCa) (1). In historically low-incidence countries such as Japan, increasing acceptance of Western diets contribute to observed increases in PCa incidence (2). However, despite substantial literature describing the potential benefits of single phytochemicals in preclinical models of PCa over six decades (3), little progress has been made in translating this knowledge into better clinical outcomes for patients. These primarily cell-culture-based studies demonstrate that phytochemicals display pleiotropic effects on multiple intracellular pathways of tumour cell growth regulation. Additional studies establish that phytochemical concentrations required to kill PCa cells are often unachievable *in vivo*, with curcumin typifying poor availability due to low oral absorption (4). Single phytochemical concentrations *in vitro* are often too low to achieve effective potency. Thus, as demonstrated in prostate cancer patients (5), single agents remain unlikely to benefit disease outcomes either through supplementation or dietary intake. In contrast, a Phase III trial of an oral capsule formulation containing freeze-dried whole foods *viz.* pomegranate, turmeric, broccoli and green tea (POMI-T^®^) has demonstrated benefit in castration resistant prostate cancer (CRPC) (6). These findings imply that phytochemicals in combination may have improved efficacy. Studies on cancer cell line models with defined compound combinations have been reported (7, 8). A randomised controlled trial of curcumin with genistein and diadzein was conducted in men undergoing prostate biopsy (9). Other clinical trials with lycopene and soy isoflavones to ‘treat’ prostate cancer were reported in 2007 and 2008, respectively, (10, 11). Major limitations are that beneficial combinations and their concentrations for maximal effect are unreported (12, 13). Based upon our literature review (14), we tested seven phytochemicals isolated from foods characteristic of Mediterranean and Asian diets for synergistic activity on cell viability of three genetically distinct prostate cancer cell lines, LNCaP, PC-3 and DU145 using a systematic high-throughput approach. Cell culture and animal studies show the effect of genistein (15), quercetin (16), indole-3-carbinol (I3C) (17), equol (18), curcumin (mouse) (19), resveratrol (20) and EGCG (21). When coupled with advanced *in silico* linkage analysis between phytochemical target proteins and PCa-promoting proteins, our data suggest that rapid identification of optimally effective phytochemical combinations based on molecular profiling of patient tumours may provide a rational approach to dietary guidance for PCa patients throughout the course of their disease from diagnosis.

## 2. Materials and methods

Cell lines (LNCaP, PC-3, DU145) were obtained from the ATCC (Manassas, VA) and identity was confirmed by short tandem repeat analysis (data not shown). Relevant characteristics of cell lines (22–33) are shown (Supplementary Table S1).

Quercetin and I3C were purchased from Sigma Aldrich (St Louis, MO) genistein, EGCG, curcumin, RS-equol, and resveratrol from Tocris Bioscience (Bristol, United Kingdom). All compounds were > 95% purity as supplied. Compound stock solutions (10 mM in dimethyl sulfoxide (DMSO) (Sigma Aldrich)) were stored at-30°C prior to use.

Cells were maintained as adherent cultures (37°C, 5% CO2, humidified atmosphere) in RPMI medium (Life Technologies, Mulgrave, Australia) supplemented with 10% foetal bovine serum, (Life Technologies). Cell viability assays (modified alamarBlue^®^ assay configured for high throughput analysis) were performed in duplicate experiments (*n* = 6 per combination for each phytochemical). Cells in logarithmic phase growth were seeded (750 cells per well, Multidrop 384 reagent dispenser (Thermo Scientific, Waltham, MA)) onto black, 384 well plates (Corning, Tewksbury, MA) and were exposed immediately to test phytochemicals or assay controls (positive control, doxorubicin (5 μM), negative control, DMSO) either by use of assay ready plates or robotic compound addition. All assays contained DMSO at 0.2%. A 10-point serial dilution of stock solutions was prepared in 384 well polypropylene plates (Greiner Bio-one Inc., Monroe, LO) and dispensed to assay plates (Hamilton STAR liquid handling robot (Reno, NV) equipped with a 384-head pin tool (V&P Scientific, San Diego, CA)). Metabolic activity was detected 72 h post-phytochemical exposure by the addition of alamarBlue reagent (Alamar Biosciences Sacramento, CA) (Multidrop Combi reagent dispenser (Thermo Scientific)) and fluorescence intensity determination (excitation 555 nm, emission 585 nm, EnSpire plate reader incorporated within a cell::explorer robotic platform, Perkin Elmer, Waltham, MA).

Cell viability relative to positive (pos) and negative (neg) assay controls were calculated from relative fluorescence unit (RFU) data as follows: % viability = (RFUsamples-RFUpos) (RFUneg-RFUpos) × 100 (Eq. 1). Positive control experiments showed complete suppression of fluorescence (data not shown). IC_50_s (Supplementary Figure S1) were calculated using a 4-parameter nonlinear regression model as follows: Y = A+(A-B)/(1 + (IC_50_/X)^C) (Eq. 2) where A = baseline response parameter, B = maximum response parameter and C = Hillslope of the dose–response curve respectively, Pair-wise combination studies were conducted using a six-point, two-fold dilution series centred on the IC_50_ concentration, where possible, in a 36-point dose matrix. Compound dilution stocks were pre-dispensed serially to assay plates (Queensland Compound Library, ECHO acoustic dispenser, Labcyte, Sunnyvale, CA). Subsequently, cells were seeded onto these assay-ready screening plates for cell viability assays. Final concentrations were: genistein, equol, I3C and resveratrol (for LNCaP and PC-3 cell lines) and EGCG, 10, 25, 50, 100, 200 μM; quercetin: 5, 12.5, 25, 50, 100 μM; curcumin: 4, 10, 20, 40, 80 μM; resveratrol (DU145): 2, 5, 10, 20, 40 μM. Synergy analysis was conducted using the BLISS and highest single agent (HSA) analysis methods (34, 35) (Supplementary Figure S2).

pTEN knockdown experiments were performed on DU145 cells using pTEN siRNA cocktail oligonucleotides (cat no: AM51331) and control siRNA oligonucleotides at a dose of 20 nM (ThermoFisher Scientific, Waltham, MA). The siRNAs were transfected into cells with Lipofectamine 2000 (Invitrogen; ThermoFisher Scientific) in a serum-free medium according to the manufacturer’s instructions. Following 48 h transfection, cells were treated with respective phytochemical combinations for 72 h. Cell viability was then assessed using a CellTiter 96^®^ AQueous One Solution Cell Proliferation Assay configured for low throughput analysis (cat. No. G3581, Promega Australia, Alexandria NSW, Australia) according to the manufacturer’s protocol. Assay absorbance was read at 490 nm on a spectrophotometer (Spectramax M2, Molecular Devices, San Jose, CA).

Western blot analysis was performed as described previously (36). Briefly, cells were washed with phosphate-buffered saline (PBS) and lysed with cold RadioImmunoPrecipitation Assay (RIPA) buffer freshly supplemented with a protease inhibitor cocktail (Sigma-Aldrich). The bicinchoninic acid (BCA) Protein Assay Kit (Beyotime Biotechnology, P0010S) was used to quantify proteins in the supernatant of cell lysates. Proteins were separated on a 10% SDS–polyacrylamide gel (NuPAGE Bis-Tris Gels, ThermoFisher) and transferred to a polyvinylidene difluoride membrane (Millipore, IPVH00010). Membranes were blocked with PBS containing 5% non-fat milk and incubated with antibodies (PTEN 1:1000; ThermoFisher United States and GAPDH antibody 1:1000; Santa Cruz biotechnologies, Texas, United States). An ECL-PLUS/Kit (Thermo, M3121/1859022) was used to detect the protein bands.

Network pharmacology analysis of phytochemical links to “causal” PCa proteins was performed as follows. Extensive searches of the literature and the databases STITCH 5.0 (Search Tool for Interactions of Chemicals and Proteins) (37), and DrugBank (38) were performed to identify human proteins targeted by the phytochemicals included in this study. The biological pathways associated with the identified protein targets were retrieved from the Kyoto Encyclopaedia of Genes and Genomes (KEGG) database (39). Pathway enrichment analysis of targets was performed using the right-sided Fisher’s exact test. Nominal *p*-values were adjusted for multiple hypothesis tests using Benjamini and Hochberg False Discovery Rate (FDR) correction. The KEGG pathways with FDR < 0.05 were regarded as significant and reported. A comprehensive platform of gene-disease associations, MalaCards (40), was used to identify the list of PCa-related genes likely associated with disease causation.

To investigate the pharmacological actions of the synergistic phytochemicals, networks showing the interactions among phytochemical compound combinations, protein targets, biochemical pathways and “causal” prostate cancer genes were constructed. Protein–protein interactions (PPIs) among targets and PCa-related genes were obtained from the comprehensive resource of validated and high-confidence predicted PPIs, “Interologous Interaction Database,” I2D version 2.9 (41). The networks were visualised in Cytoscape (42). All statistical analyses were performed in R (v3.6.3).^1^

## 3. Results

Curcumin was the most potent single agent in all cell lines (apparent IC_50_ 9 to 12 μM) (Supplementary Figure S1). Resveratrol showed equivalent potency in DU145 cells and was ~10-fold lower in LNCaP or PC-3 cells. I3C potency was similar in DU145 and LNCaP cells but minimal in PC-3 cells. Overall, potency was weak (6/21 combinations IC_50_ < 25 μM). DU145 cells were the most sensitive (5/7 compounds IC_50_ < 100 μM).

Dual phytochemical treatment analysis by Bliss or HSA models showed cell-line-dependent responses (Table 1; Supplementary Table S2; Supplementary Figure S3). The most synergistic duos (Bliss equation) were I3C/quercetin (LNCaP and DU145) and I3C/genistein (PC-3). Maximal antagonistic response occurred with quercetin/genistein (LNCaP), quercetin/EGCG (PC-3) and high concentrations of I3C/EGCG or EGCG/curcumin (DU145) (Supplementary Table S2). Some combinations were additive at most concentrations, notably equol/EGCG (LNCaP and PC-3) (Supplementary Figures S3A,B) and curcumin/equol (DU145) (Supplementary Figure S3C).

**Table 1:** Phytochemical combinations with maximum synergy in each cell line.

	LNCaP	PC-3	DU145
Quercetin	I3C 0.61^1^, 12.5/100^2^	I3C 0.38, 12.5/200	I3C 0.39, 25/50
Genistein	I3C 0.38, 50/100	I3C 0.48, 50/200	Resveratrol 0.31, 25/50
I3C	Quercetin 0.61,100/12.5	Genistein 0.48, 200/50	Quercetin 0.39, 50/25
EGCG	Genistein 0.28, 100/25	Genistein 0.39, 100/50	Resveratrol 0.35, 100/50
Curcumin	I3C 0.35, 20/100	I3C 0.42, 20/100	I3C 0.35, 20/50
Equol	I3C 0.33, 100/100	I3C 0.31, 100/200	Resveratrol 0.38, 25/100
Resveratrol	I3C 0.34, 50/100	I3C 0.24, 50/100	Equol 0.38, 100/25

^1^Potency of synergistic response. ^2^Phytochemical concentration (μM) in each pair at which maximum synergy was measured. First number refers to phytochemical in column 1, while the second number refers to the phytochemical in cell-line-specific columns.

The HSA model accentuates synergistic responses, while the Bliss model accentuates antagonism at high concentrations of curcumin and I3C. For combinations with relatively weak synergy or antagonism, the two models give different results. The two models gave inconsistent synergy results for resveratrol/genistein (LNCaP) (Supplementary Figure S3A), combinations of quercetin with genistein, curcumin or resveratrol and combinations of resveratrol with EGCG or curcumin (PC-3) (Supplementary Figure S3B) and genistein/quercetin (DU145) (Supplementary Figure S3C). Despite this heterogeneity, all combinations show synergy in at least one model.

**Figure 1:**
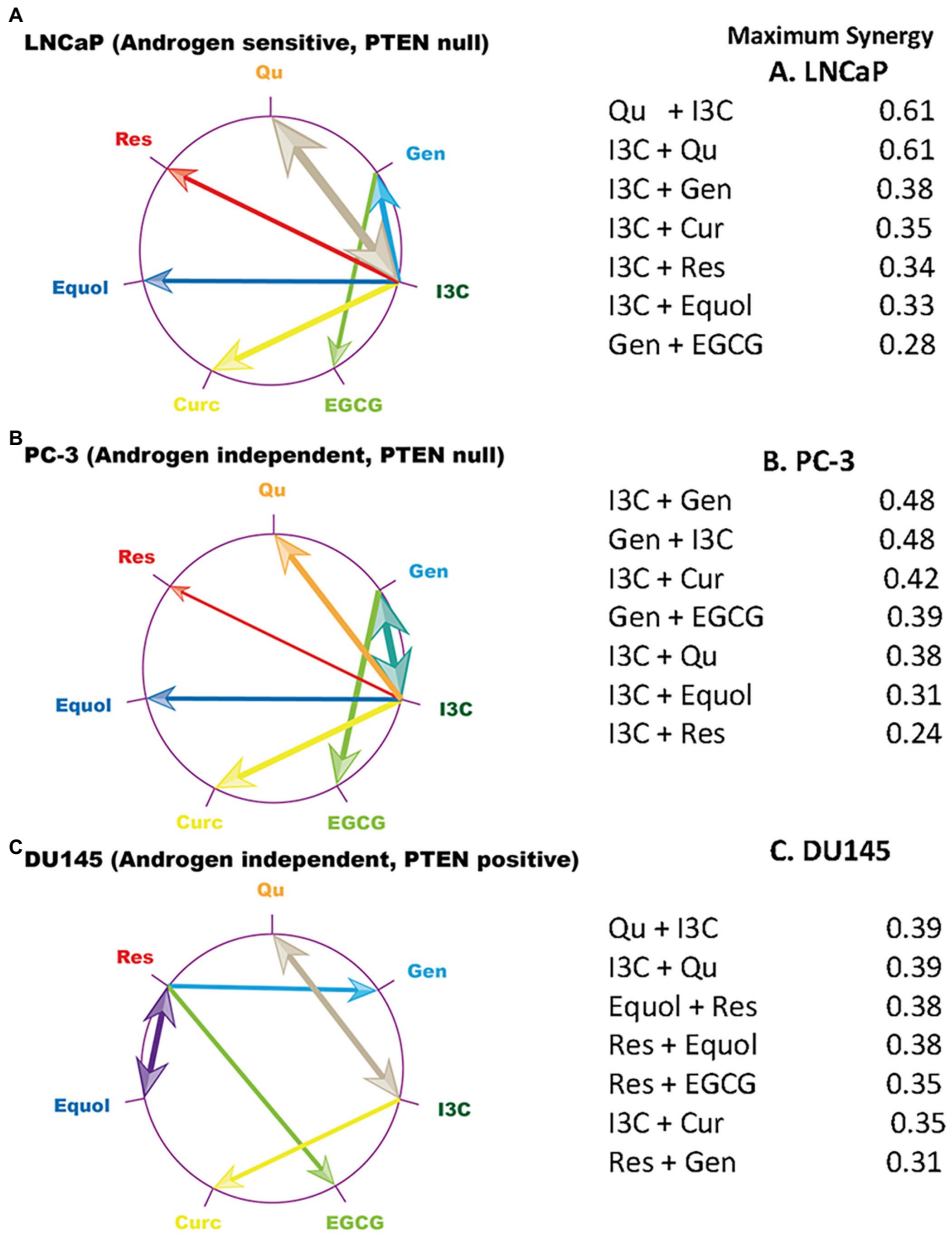
Maximum synergy signatures of prostate cancer cell line models. The compound which showed the maximum synergy at any concentration ratio in combination with each phytochemical on the edge of the circle is linked to that phytochemical by an arrow. Arrow width is scaled to represent the size of the maximum synergistic response which is tabulated. Data plotted were generated from the Bliss model (Supplementary Figures S3A–C). Qu, quercetin; Gen, genistein; I3C, indole 3 carbinol; EGCG, epigallocatechin gallate; Curc, curcumin; Res, resveratrol. (**A**) LNCaP cells, (**B**) PC-3 cells, (**C**) DU145 cells.

Higher-order relationships between maximally synergistic phytochemical combinations (Bliss results) were identified using polygonograms (43) (Figure 1). These results revealed unique patterns of maximum synergy in the three cell lines. I3C is maximally synergistic with both quercetin and curcumin in all three cell lines. However, resveratrol contributes to maximum synergy with equol, EGCG and genistein in DU145 cells, while having no maximum synergy relationships with any phytochemical in LNCaP and PC-3. The synergy pattern is strongly conserved between LNCaP and PC-3 cells, despite these two cell lines having different androgen status (Supplementary Table S1). Conversely, maximal synergistic relationships between phytochemical pairs in the DU145 cell line are unique, despite having a similar androgen status to PC-3 cells. Similarly, neither p53 status, inflammatory marker status (NF-κB or IL-6 expression), nor p27/Kip1 status (Supplementary Table S1) explains the observed patterns. In contrast, these patterns are related to pTEN status, with LNCaP and PC-3 cells being pTEN-null while DU145 is pTEN-positive (Figure 1).

**Figure 2:**
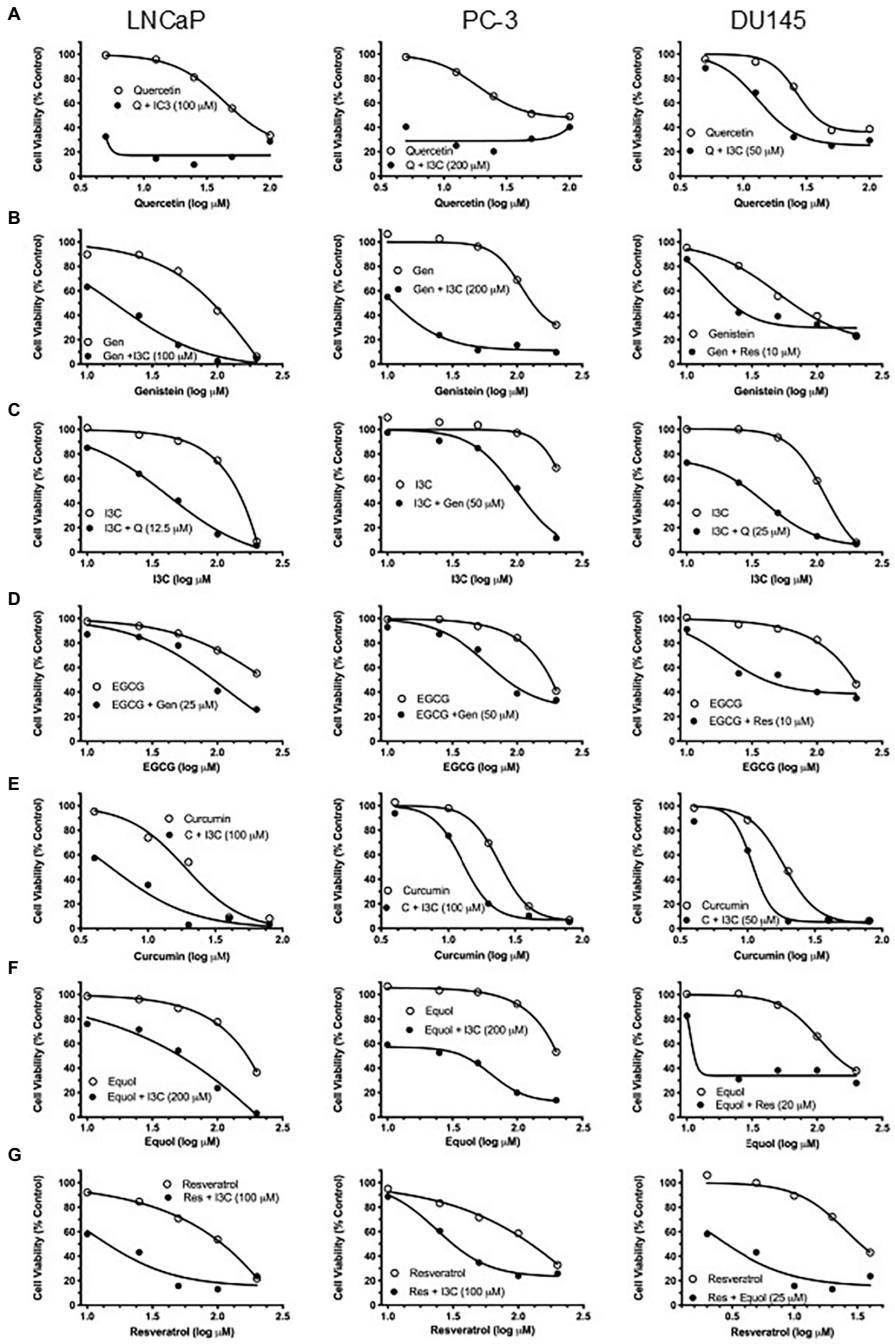
Effect of maximally synergistic combination on PC potency in each cell line. Cell viability of single-agent phytochemical (open symbols) and maximally synergistic dual combination (closed circles) for each phytochemical in each cell line. Identity and concentration (μM) of com-bination phytochemical is labelled in each graph. **(A)** Quercetin, **(B)** genistein, **(C)** I3C, **(D)** EGCG, **(E)** curcumin, **(F)** equol, **(G)** resveratrol.

**Figure 3:**
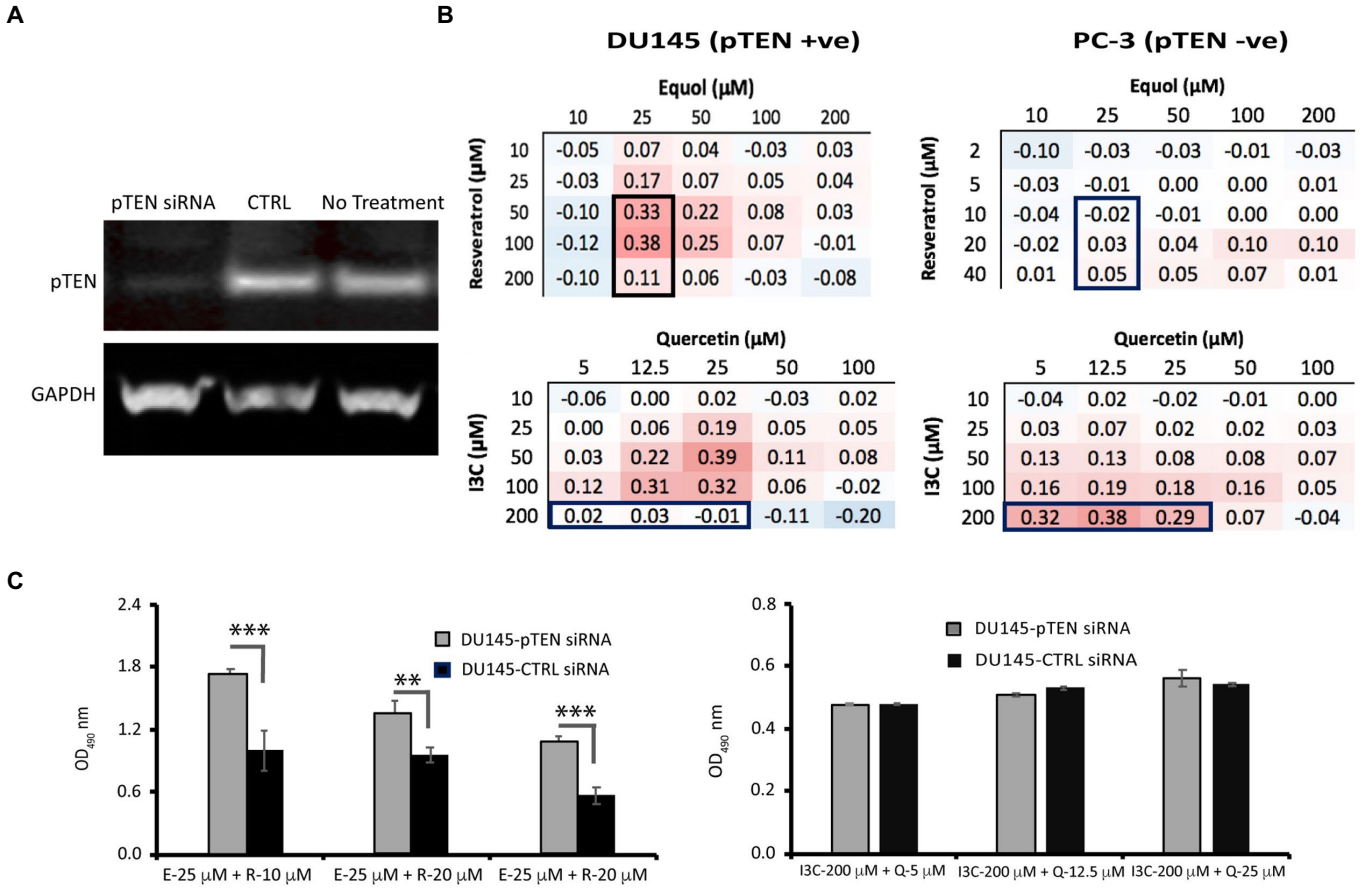
Effect of pTEN knockdown on resveratrol/equol synergy and I3C/quercetin synergy in DU145 cells. **(A)** DU145 cells were depleted of pTEN protein expression using siRNA knockdown as confirmed by Western blot analysis relative to the housekeeping protein glyceraldehyde phosphate dehydrogenase (GAPDH). **(B)** To test whether synergy in DU145 (pTEN +ve) cells was dependent on pTEN expression, three synergistic combinations of resveratrol/equol in DU145 cells but not in PC-3 (pTEN-null) cells (top panel boxed) and three combinations of I3C and quercetin that were not synergistic in DU145 cells but were in PC-3 cells (bottom panel boxed) were tested for their effect on cell viability by MTT assay in DU145 knockdown cells relative to wild-type DU145. **(C)** Cell viability post-pTEN-siRNA-mediated knockdown in DU145 cells 48 h. after transfection with pTEN siRNA (20 nM). Data are mean ± SD of triplicate determinations. ***p < 0.001; **p < 0.01 versus control (unpaired Student’s *t*-test).

However, to reveal the effect of maximally synergistic combinations on the potency of cell killing, the dose–response for each phytochemical with its maximally synergistic partner in each cell line (Table 1) was plotted relative to the dose–response of the phytochemical. Quercetin/I3C (Figure 2) is the most potent synergistic combination in LNCaP (IC_50_ < 4.5 μM quercetin with 100 μM I3C) and in PC-3 cells (IC_50_ < 4.5 μM quercetin with 200 μM I3C). In contrast, despite displaying lower overall synergy than I3C/quercetin (Table 1), resveratrol/equol was most potent in DU145 cells (IC_50_ ~ 2.8 μM resveratrol with 25 μM equol). Overall, the greatest synergistic impact on potency in combination with other phytochemicals with 15/21 (71%) maximally effective combinations involved I3C. I3C with quercetin or curcumin was synergistic in all cell lines. In combination, I3C/curcumin showed greater potency than curcumin alone, with IC_50_ of the combination improving by ~10-fold in LNCaP cells and 2-fold in PC-3 and DU145 cells. Our synergy data predicts that the pattern of the maximum synergy of dual combinations of each of the seven phytochemicals is dependent on cellular genotype. Our hypothesis was that pTEN loss, a common feature in castrate-resistant prostate cancer patients and seen in AR-positive LNCaP and AR-negative PC-3 cells, reduces the influence of resveratrol on maximum synergy in DU145 cells. To test this, pTEN knockdown experiments were performed in DU145 cells (Figure 3A) followed by assaying the effect of knockdown on cell viability of the maximally-synergistic combination in DU145 cells, resveratrol/equol. In addition, to determine whether pTEN knockdown was able to improve synergy in DU145 cells of the dominant synergistic combination seen in pTEN-null cell lines (I3C/quercetin), the maximally synergistic concentrations of these two phytochemicals in PC-3 cells were also assayed in pTEN-knockdown DU145 cells (Figure 3B). pTEN knockdown in DU145 cells had no effect on the potency of I3C and quercetin combination. However, the potency of resveratrol and equol was significantly reduced in pTEN siRNA-treated DU145 cells (Figure 3C). This suggests that equol and resveratrol synergistic action is predominantly dependent on the presence of pTEN in DU145 PCa cells.

**Figure 4:**
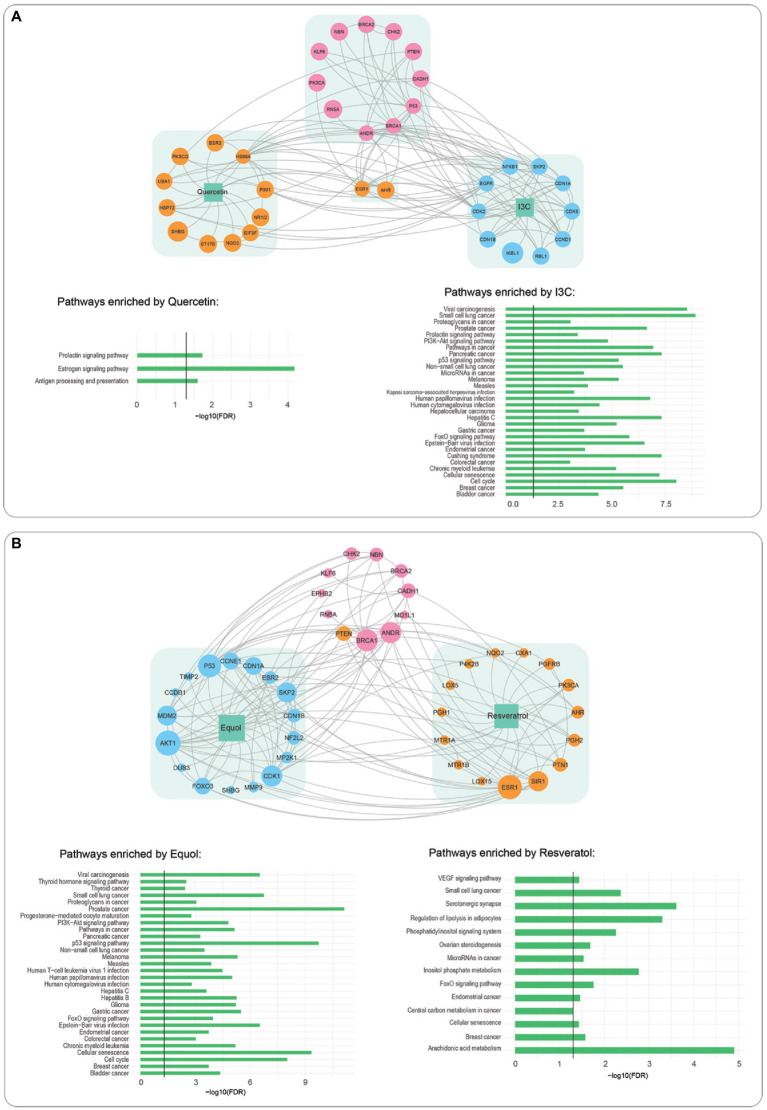
Interactions of phytochemical interactome with prostate cancer driver proteins and pathway enrichment analysis. **(A)** I3C interactome (blue), quercetin interactome, (orange). **(B)** Equol (blue) resveratrol (orange). Linkages to prostate cancer interactome (pink) are indicated by grey lines, Bar graphs indicate pathways significantly enriched by each phytochemical.

Relationships between the most potent phytochemical combination for each cell line and protein targets known to promote PCa were examined using a network pharmacology analysis of known links of each maximally effective phytochemical, both alone and in combination, to human protein targets and subsequently to known PCa protein targets. Quercetin alone had direct interactions with 13 proteins. Two (ESR1 and AHR) shared direct interactions with I3C, which also linked to 10 additional proteins (Figure 4A). Pathway enrichment analysis indicated that the quercetin interactome targeted three biological pathways. Two were associated with hormone signaling. In contrast, the 13 proteins in the I3C interactome were strongly represented in 30 pathways; 12 organ-specific cancers, nine cancer-related biochemical pathways and six associated with infectious disease. When linkages of the combined interactomes of quercetin and I3C to proteins known to potentiate PCa were examined, 11 proteins were directly linked to the phytochemical targets. The majority were tumour suppressor proteins or transcription factors.

Network pharmacology analysis of equol/resveratrol, the most effective DU145 combination (Figure 4B), identified a 17-protein interactome for equol and a 15-protein interactome for resveratrol. Interestingly only six of these proteins were common to either the quercetin or I3C interactomes. Despite these differences, pathway enrichment analysis showed that 25/30 pathways the equol interactome was associated with were identical to the I3C interactome (Figure 4A). Six/14 resveratrol-enriched pathways were shared with equol. The most significantly enriched resveratrol pathway was arachidonic acid metabolism, a pathway well known to promote PCa. The equol/resveratrol interactome was directly linked to 11 PCa-potentiating proteins, seven of which were also directly associated with the quercetin/I3C combination.

The effect of the major genotypic alterations in each of the three cell lines LNCaP, (AR positive, pTEN null, p53 positive), PC-3 (AR negative, pTEN null, p53 negative), and DU145 (AR negative, pTEN positive, p53 positive) on the PCa protein interactome for each cell line was also examined (Table 2; Supplementary Figure S4). pTEN loss in LNCaP cells did not affect quercetin/I3C interactions with the remaining 10 PCa-promoting proteins (Supplementary Figure S4A). In PC-3 cells, the loss of all three driver genes, resulted in the further loss of one PCa-promoting protein, RN5A, from the quercetin/I3C network (Supplementary Figure S4B). The loss of AR in DU145 cells also resulted in the loss of the RN5A interaction in the equol/resveratrol linkage map (Supplementary Figure S4C).

**Table 2:** Cell-line-dependent linkages of most potent synergistic phytochemical combinations to prostate cancer genes.

Cell line phytochemicals	LNCaP quercetin/I3C	PC-3 quercetin/I3C	DU145 equol/resveratrol	Role in PCa^1^
Gene link	BRCA1	BRCA1	BRCA1	Tumour suppressor, DNA damage repair, upregulated by phytochemicals
	BRCA2	BRCA2	BRCA2	Tumour suppressor, DNA damage repair, upregulated by phytochemicals
	CADH1	CADH1	CADH1	Epithelial Adhesion molecule, invasion suppressor, upregulated by resveratrol (DU145)
	CHK2	CHK2	CHK2	Tumour suppressor, Ser/Thr kinase, checkpoint-mediated cell cycle arrest, DNA Damage repair, induces apoptosis in response to double strand breaks
	KLF6	KLF6	KLF6	Tumour suppressor, Kruppel-like zinc finger transcription factor, phytochemicals induce expression
	NBN	NBN	NBN	DNA damage repair protein. Over-expressed in PCa. Repair is often inaccurate
	PK3CA	PK3CA	-	Catalytic subunit of PI3 kinase. Overactivated in cancers. Phytochemical inhibit activity
	RN5A	-	-	Ribonuclease L, antiviral endonuclease, interaction between the androgen receptor mediates cross-talk between the interferon and androgen signalling pathways
	ANDR	-	-	Androgen-dependent transcription factor. Aberrantly expressed in PCa
	p53	-	-	Tumour suppressor, Transcription factor. Functions in growth arrest, DNA damage repair and apoptosis induction
	-	-	pTEN	Tumour suppressor, dual specificity protein and lipid phosphatase, blocks PI3Kinase signaling
	-	-	EPHB2	Tumour suppressor, receptor tyrosine kinase, Mutated in PCa
	-	-	MD1L1	Essential component of the mitotic spindle assembly checkpoint

^1^Data obtained from MalaCards.

## 4. Discussion

I3C is identified here as a major promoter of synergy featuring in 12/18 (67%) maximally synergistic combinations (Table 1). I3C is particularly prominent in the pTEN-null cell lines LNCaP and PC-3 (83% of combinations) compared to DU145 cells (33% of combinations). In addition to its function inducing pTEN expression (44), I3C also indirectly reactivates the tumour-suppressive function of pTEN through inhibition of the ubiquitin ligase WWP1 (45), a mechanism that explains its effect in pTEN-positive cancer cells. I3C is also reported to have suppressive effects on the phosphorylation of AKT kinase in PC-3 cells resulting in failure to activate the AKT/PI3K pathway (46). Thus, it is likely that I3C compensates for pTEN loss by this mechanism, at least in this cell line.

Every compound showed synergy with multiple phytochemicals; however, the potency of cell killing with maximally synergistic combinations varied significantly for each phytochemical and between cell lines (Figure 2). In DU145 cells, despite all combinations having very similar maximum synergy (Table 1), resveratrol/equol was a substantially more potent combination than any other (Figure 2). The models used to identify maximum synergy in this study focus primarily on efficacy without being able to account for combination potency. The recently-published quantitative method (MuSyC) (47) takes both the efficacy and potency of drug combinations into account and may provide additional insight into the most appropriate phytochemical combinations for any particular tumour cell genotype. This approach may be particularly useful when considering the limitation on understanding the real benefits of phytochemicals due to the discrepancy between effective concentrations *in vitro* and achievable concentrations *in vivo* that is seen with some, but not all, combinations.

Pattern analysis revealed a strikingly similar profile in pTEN-null LNCaP and PC-3 cell lines, despite their dramatically different androgen responsiveness, in contrast to pTEN-positive DU145 cells (Figure 1). Thus, the responsiveness of tumour cells to dual phytochemicals in these cellular models, is independent of androgen sensitivity, since the synergy profile of PC-3 and LNCaP cells is similar. The mechanism by which the phytochemical combinations help overcome anti-androgen resistance is not known; however, there are several ways that antiandrogen resistance is proposed to occur (48). For example, induction of constitutively active androgen-independent splice variants of AR such as AR-V7 can lead to AR signaling in the absence of androgen. Secondly, androgen receptor bypass signaling can occur. There is evidence that in animal models with acquired resistance to enzalutamide, the glucocorticoid receptor (GR) is upregulated and enzalutamide resistance is dependent on GR expression in cell lines. GR activates a restricted set of AR target genes, which are presumed to be sufficient to promote growth. Thirdly, complete androgen receptor independence can occur in subsets of cells that have a neuroendocrine phenotype, characterised by loss of tumour suppressors pTEN, Rb and p53. While there is evidence that DU145 and PC-3 cells express GR there is no publicly available information on the effect of the phytochemical combinations identified here on its expression. The data presented here provides a model whereby these potential mechanisms can be explored.

In contrast to AR status, responsiveness appears to reflect the pTEN status of the cell lines. The results of our pTEN knockdown experiments in DU145 cells (Figure 3) confirm that reducing pTEN expression suppresses the synergistic effect of maximally synergistic resveratrol/equol combinations without affecting response to I3C/quercetin. This finding validates the principle in the case of pTEN expression in DU145 cells, that the molecular profile of a prostate cancer cell selectively regulates the responsiveness of these cells to phytochemicals in combination. Knocking down pTEN gene expression, was equivalent to converting DU145 to a PC-3 phenotype with respect to the cell’s responsiveness to resveratrol/equol. The result raises the intriguing possibility that patients with pTEN loss may benefit from a different dietary phytochemical intake than pTEN-positive patients. pTEN-loss through mutation has been reported in 44% of castration resistant prostate cancer cases (49) representing up to 70% of men diagnosed with prostate cancer, influencing both cancer initiation and metastatic PCa recurrence. Therefore, though additional studies are required to establish this, pTEN status may be a useful indicator in providing targeted dietary advice to patients.

Our network pharmacology approach suggests that, despite individual phytochemicals having diverse interactomes, the most potent phytochemical combinations target a similar subset of proteins known to be associated with PCa progression (Figure 4) and that modulating these proteins is sufficient to produce the most potent synergistic response in suppressing cell viability. Most of these proteins are well known tumour suppressors or transcription factors. A core subset of six PCa proteins are linked with the most potent phytochemical combination in all cell lines regardless of cancer cell genotype, with seven other proteins identified as cell-line dependent links (Table 2). While there is no information in the literature relating to the effect of these phytochemical combinations on the expression of the genes identified here, BRCA1 and BRCA2 are known to be induced in both LNCaP and DU145 cells with I3C-mediated toxicity being dependent in part on BRCA1 and BRCA2 (50). A combination of I3C and genistein resulted in greater induction than I3C alone. These data are consistent with the identification of BRCA1 and BRCA2 being important in phytochemical-mediated cytotoxicity as identified in our network pharmacology studies. The role of phytochemicals in chemoprevention, though well discussed in the scientific literature is yet to be definitively determined, due, in part, to the complexity of both the number of phytochemicals known to have *in vitro* benefit and the imprecision of our understanding of the genetic targets of common phytochemicals. The finding here, that a limited number of genes critical for genome integrity may be the targets of phytochemical treatment could focus research in the field and provide potential pathways to study patients showing pre-neoplastic lesions that often precede the onset of tumorigenesis.

In contrast to the equol/resveratrol combination, the knockdown of pTEN was insufficient to improve the potency of the I3C/quercetin combination in DU145 cells at concentrations that are maximally synergistic in PC-3 cells (Figure 3). This suggests that the efficacy of I3C and quercitin is not predominantly dictated by pTEN levels. Our network analysis (Figure 3B) shows that pTEN is a direct target of resveratrol and an indirect target of I3C or quercetin. Since PC-3 cells are both AR and pTEN-null and the DU145 knockdown has a similar phenotype with respect to the expression of these two genes, the remaining interactions in PC-3 cells (Supplementary Figure S4B), such as ESR1, may be regulating the synergy of I3C and Quercetin combinations rather than pTEN.

Importantly, we have shown that the variation in individual synergistic response is a genetic trait. Again, to our knowledge, this has not been shown earlier and has potentially great promise in understanding how synergy and antagonism occur. Specifically, if there is a genetic basis for phytochemical synergy, this could explain why individual responses to dietary intervention vary from person to person. Maximally synergistic phytochemical concentrations approached physiologically relevant concentrations equating to dietary intake. Therefore, our study may also be helpful in defining a diet of whole foods based on phytochemical concentrations. A 30-day intake of 4 g/d curcumin resulted in blood levels up to 11.9 μM (51), while 30 days of 80 mg isoflavones increased plasma metabolite daidzein by 0.31 μg/ml and genistein by 0.52 μg/ml isoflavones (52). Circulating quercetin concentrations of 1.0 μM – 4.0 μM were obtained following 50 mg daily dosing (53), while 5 g resveratrol resulted in blood concentration of 2.3 μM (54).

Application of high throughput approaches has its limitations in that the number of experiments needed to determine synergy grows exponentially with the number of combinations screened. Recent advances in mathematical modelling have identified a way of predicting the effect of multiple combinations of drugs based on measurements of drug pairs (55), thus reducing the number of high throughput experiments required to a manageable level and expanding the capacity of the approach described here to include more phytochemical combinations. Together with the network-based analytical approaches described in our study, the use of such predictive algorithms may make a precision medicine approach possible, *viz.* the prediction of the most effective combination of phytochemicals to include in a patient dietary plan based on a genetic, transcriptomic, proteomic and lipidomic molecular profile of a patient tumour taken at biopsy or on surgery. The approach may also be useful over the course of patient treatment by providing molecular-profile-guided dietary advice derived from the analysis of liquid biopsies.

## 5. Conclusion

In summary, our findings provide a systematic analysis of synergy in three genotypically distinct prostate cancer cell lines, demonstrating that optimal phytochemical combinations are dependent on the molecular profile of tumour cells and provide a pathway for the development of patient-specific dietary advice based on tumour molecular profiling.

## Data availability statement

The raw data supporting the conclusions of this article will be made available by the authors, without undue reservation.

## Author contributions

CG, PdS, FV, and KS: conceptualization. SF, TF, and GA: methodology. SF and FV: software. SF, TF, GA, and MS: validation. TF, GA, SF, and FV: formal analysis. CG, SF, and KS: investigation. TF, GA, FV, DM, PdS, and KS: resources. TF, GA, SF, FV, and KS: data curation. CG, SF, TF, GA, DM, AS, SF, FV, and KS: writing – original draft preparation. CG, SF, AF, GA, MS, DM, AS, JC, JB, PdS, FV, and KS: writing – review and editing. CG, TF, GA, SF, FV, and KS: visualization. DM, JC, JB, PdS, and KS: supervision. CG and PdS: project administration. JB, DM, and PdS: funding acquisition. All authors contributed to the article and approved the submitted version.

## Funding

This project was supported by an Australian Research Council (ARC) PhD Scholarship award to CG.

## Conflict of interest

The authors declare that the research was conducted in the absence of any commercial or financial relationships that could be construed as a potential conflict of interest.

## Publisher’s note

All claims expressed in this article are solely those of the authors and do not necessarily represent those of their affiliated organizations, or those of the publisher, the editors and the reviewers. Any product that may be evaluated in this article, or claim that may be made by its manufacturer, is not guaranteed or endorsed by the publisher.

## Footnotes

^1^ http://www.r-project.org/
