# Supplementary material for: Anti-cancer potential of synergistic phytochemical combinations is influenced by the genetic profile of prostate cancer cell lines
Source: Front Nutr. 2023 Mar 7;10:1119274. doi: 10.3389/fnut.2023.1119274 (PMC10029761; doi:10.3389/fnut.2023.1119274)
Supplement: Supplementary file 1 [file Data_Sheet_1.docx]

Supplementary Material

Title

**Anti-cancer potential of synergistic phytochemical combinations are influenced by the genetic profiles of prostate cancer cell lines**

**Authors**

**Carol A. Gano^1^, Shadma Fatima^1, 2 ,3,^*, Timothy W. Failes^4^, Greg M. Arndt^4,5^, Mila Sajinovic^2^, David Mahns^1^, Ahmad Saedisomeolia ^6^, Jens R. Coorssen^7^, Joseph Bucci^8^, Paul de Souza^1, 2,^, Fatemeh Vafaee^3,^ and Kieran F. Scott ^1, 2,^***

^1^ School of Medicine, Western Sydney University, Campbelltown, NSW 2560, Australia. CAG

^2^ Ingham Institute of Applied Medical Research, Liverpool, NSW 2170, Australia. MS,

^3^ School of Biotechnology and Biological Sciences, UNSW Sydney NSW 2052, Australia. FV,

^4^ ACRF Drug Discovery Centre, Children’s Cancer Institute, Lowy Cancer Research Centre, UNSW Sydney, Sydney, NSW, Australia.

^5^ School of Clinical Medicine, UNSW Medicine & Health, UNSW Sydney, Sydney, NSW, Australia.

^6^ School of Human Nutrition, McGill University, Ste-Anne-de-Bellevue, Quebec, H9X 3V9, Canada. AS,

^7^ Departments of Health Sciences and Biological Sciences, Faculties of Applied Health Science, and Mathematics and Science, Brock University, St Catharine’s, Ontario, Canada. JRC

^8^ St George Hospital Clinical School, UNSW, Kogarah 2217 NSW, Australia. JB,

***** Co-correspondence: [s.fatima@westernsydney.edu.au](mailto:s.fatima@westernsydney.edu.au)

***** Correspondence: [kieran.scott@westernsydney.edu.au](mailto:kieran.scott@westernsydney.edu.au)

SU**pplementary Tables and FiGURES.**

Table S1. Detailed characteristics of cancer cell lines

| Cell line | LNCaP | PC-3 | DU145 | References |
| --- | --- | --- | --- | --- |
| Origin | 50 yr old Caucasian (1977) supraclavicular lymph node | 62 yr old Caucasian (1979) lumbar vertebra Grade IV Disease | 60 yr old Caucasian (1975), Dura mater | [[22](#_ENREF_22), [23](#_ENREF_23)] |
| Cytogenetics | Modal chromosome number 76 (range 33-91) also reported as : Modal number 76 to 91 | Modal chromosome number 65 (passage 58), Y-chromosome negative | Modal chromosome number 64, passage 90; Y- chromosome positive (range 46-143, passage 57) | [[22](#_ENREF_22), [24](#_ENREF_24)] |
| Tumorigenicity | Low | High | Moderate | [[22](#_ENREF_22), [25](#_ENREF_25)] |
| Metastatic potential | Low | High | Moderate | [[26](#_ENREF_26)] |
| Androgen status | Sensitive; AR positive (T788A), receptor binds progesterone and estradiol | Resistant; AR negative | Resistant; AR negative | [[27](#_ENREF_27)] |
| PSA status | Positive | Negative | Negative | [[28](#_ENREF_28)] |
| p53 status | Positive; wild-type; over-expressed | Negative; hemizygous chromosome 17p base pair deletion codon 138 frame-shift stop at codon 169 position | Positive; 2  Condon mutations  274 pro > leu 223 val > phe | [[29](#_ENREF_29)] |
| Constitutive NF-kB status | Negative | Positive | Positive | [[30](#_ENREF_30)] |
| IL-6 expression status | Does not produce IL-6. Responsive to IL-6 stimulation. | Increased expression and secretion due to constitutive NF-kB activation. | Increased expression and secretion due to constitutive NF-kB activation. | [[31](#_ENREF_31), [32](#_ENREF_32)] |
| p27KIP1 status | Expression is inversely correlated with skp2 (ubiquitin ligase) expression skp2 is NOT regulated by Akt | Skp2 is the main determent in PI3K/Akt dependent regulation of p27Kip1 | Skp2 is the main determent in PI3K/Akt dependent regulation of p27Kip1 | [[33](#_ENREF_33)] |
| pTEN status | Null | Null | Positive | [[33](#_ENREF_33)] |

Table S2. Maximum synergy and antagonism for dual combination phytochemicals on prostate cancer cell lines for the Bliss model


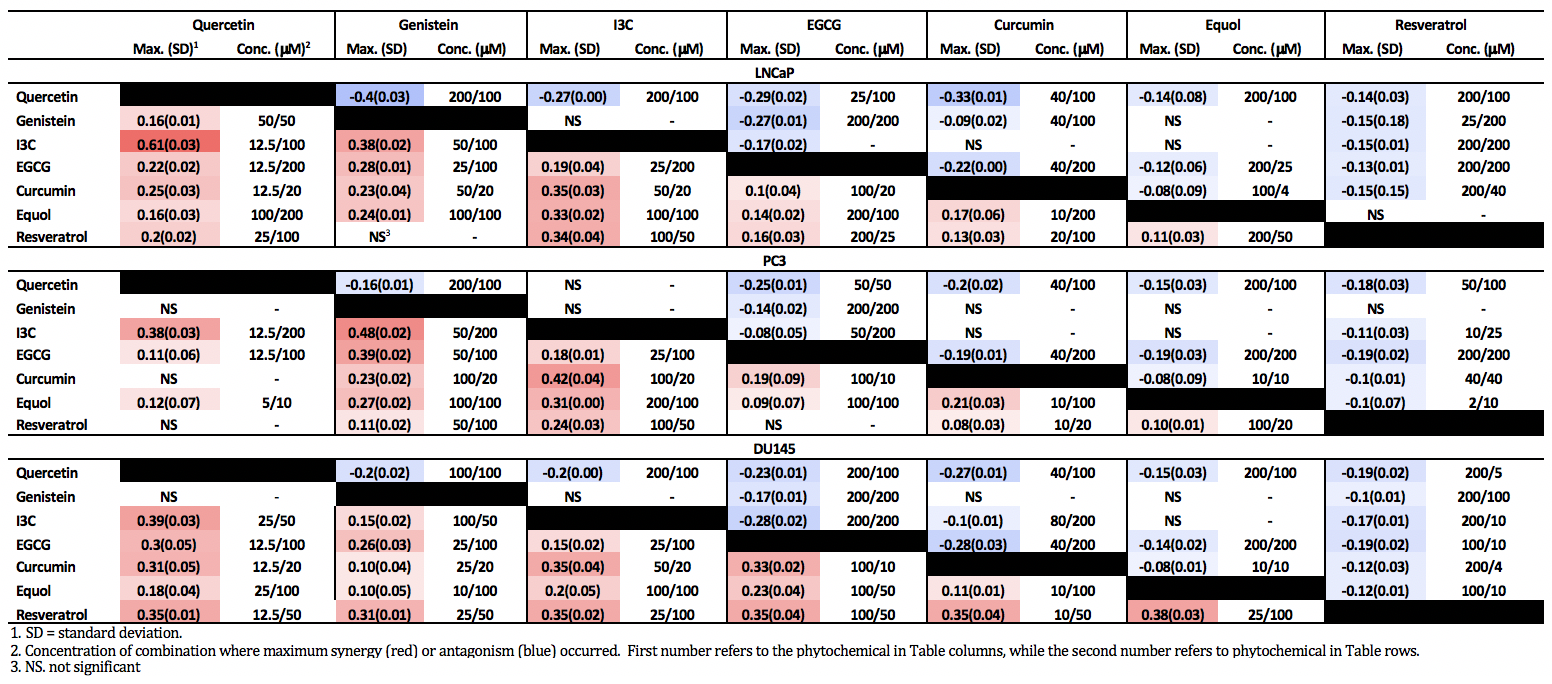


**Figure S1.**

Figure S1. Single-agent-phytochemical IC_50_ determination. Data were fit to a 4-parameter nonlinear regression model as described in Materials and Methods. Baseline response term (A) was fixed at 0% and the maximum response term (B) fixed at 100% with the following exceptions: quercetin (DU145 and PC-3), (A) and (B) unfixed; genistein (DU145) (A) fixed (0%); resveratrol (DU145), (B) fixed (100%). R^2^ values for the curve fits were > 0.95. Dose response model fit ten individual % viability data points per concentration.

**Figure S2.**

**
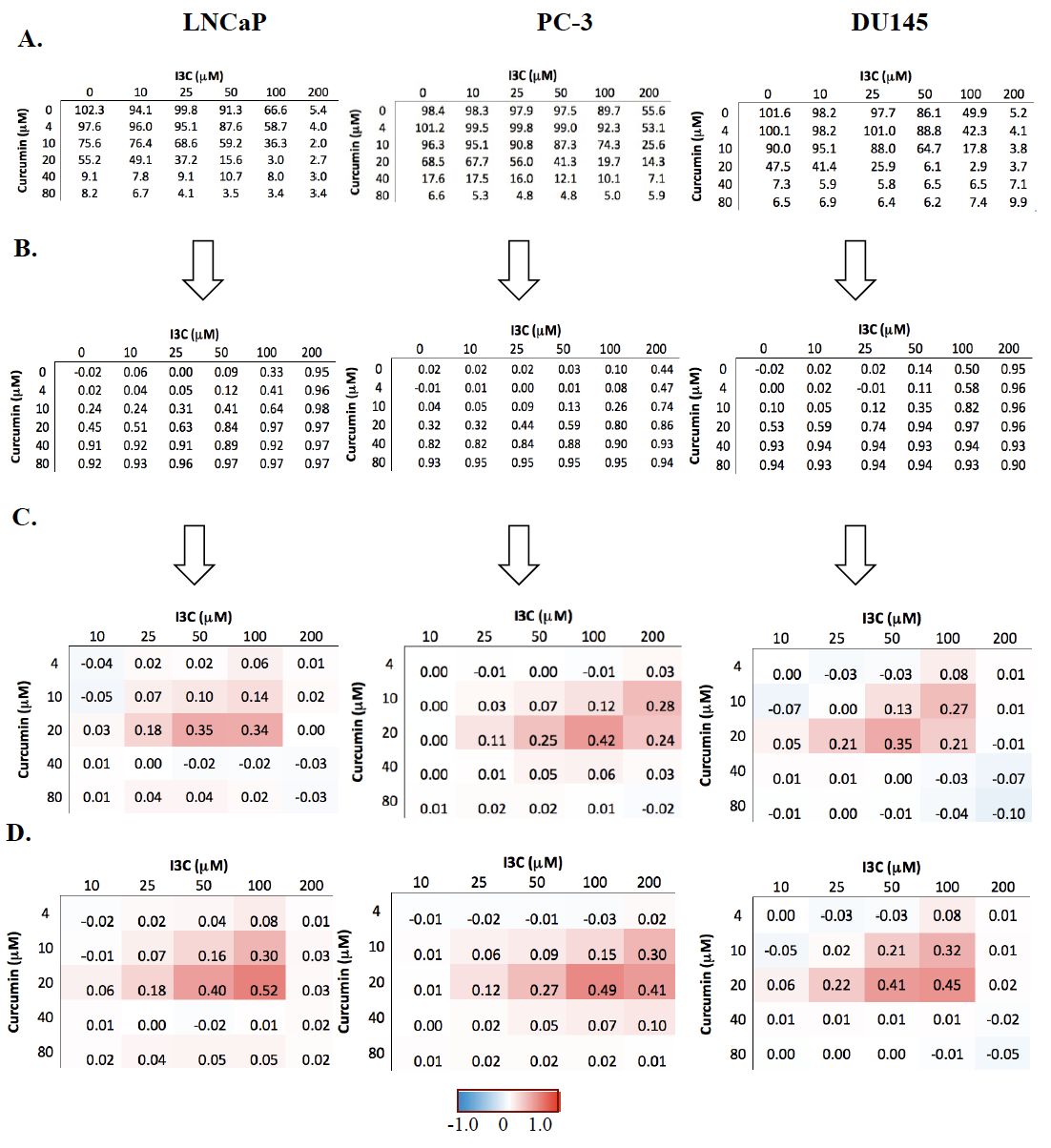
**

Figure S2. Synergy Profile. A. Mean % cell viability B. Transformed data to mean fractional growth inhibition. C. Synergy calculated using the BLISS model. D. Synergy calculated using the HSA model. Heat map plotted between -1.0 (antagonism, blue), 0 (additivity, white) and 1.0 (synergy, red). Synergy or antagonism relative to additivity was evaluated from the estimated variance across the 75 means (25 from each biological replicate) for each cell line.

**Figure. S3A.**

.**
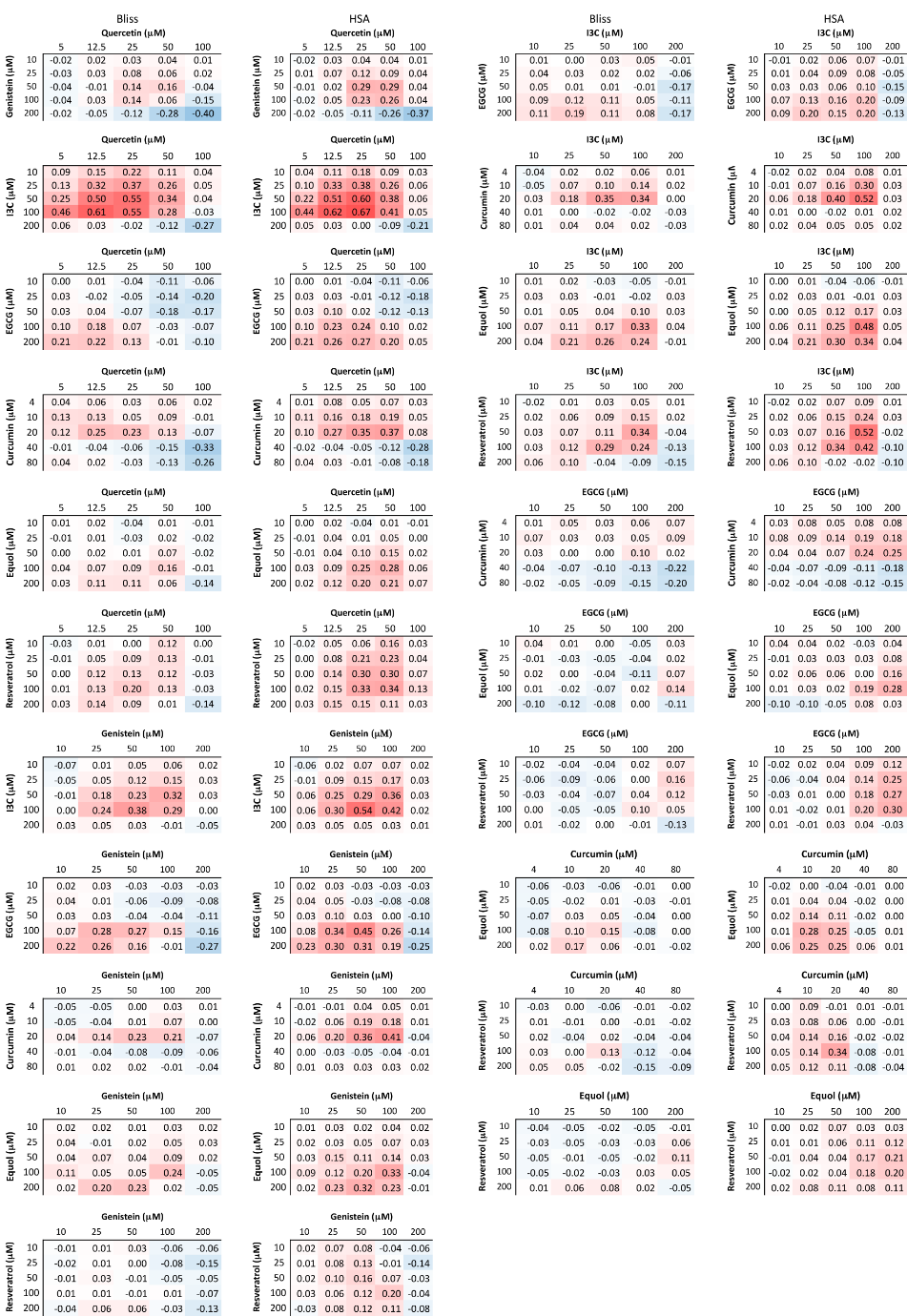
**

**Figure S3B.**


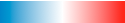


-1.0 0 1.0


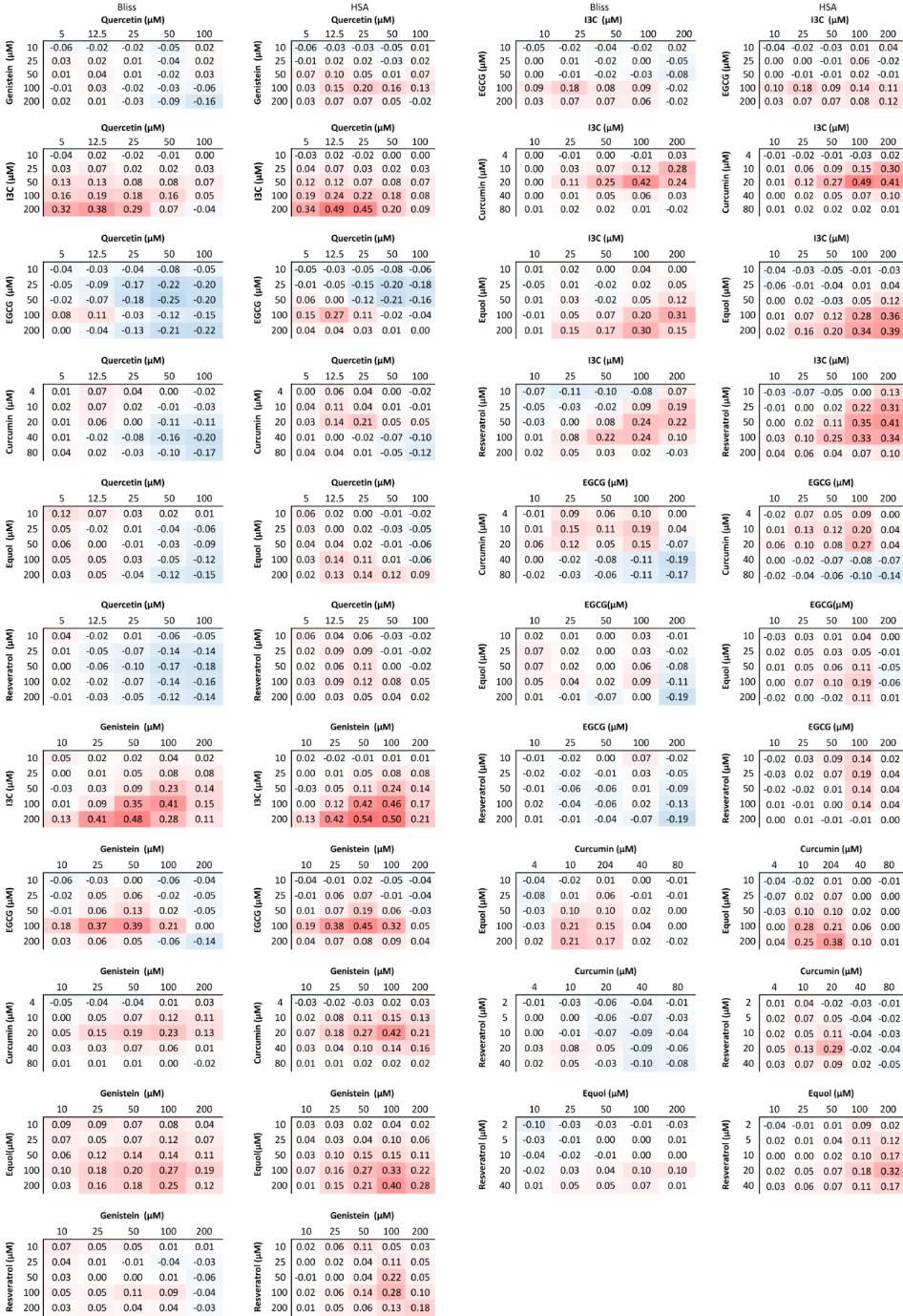


**Figure S3C.**


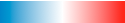


-1.0 0 1.0


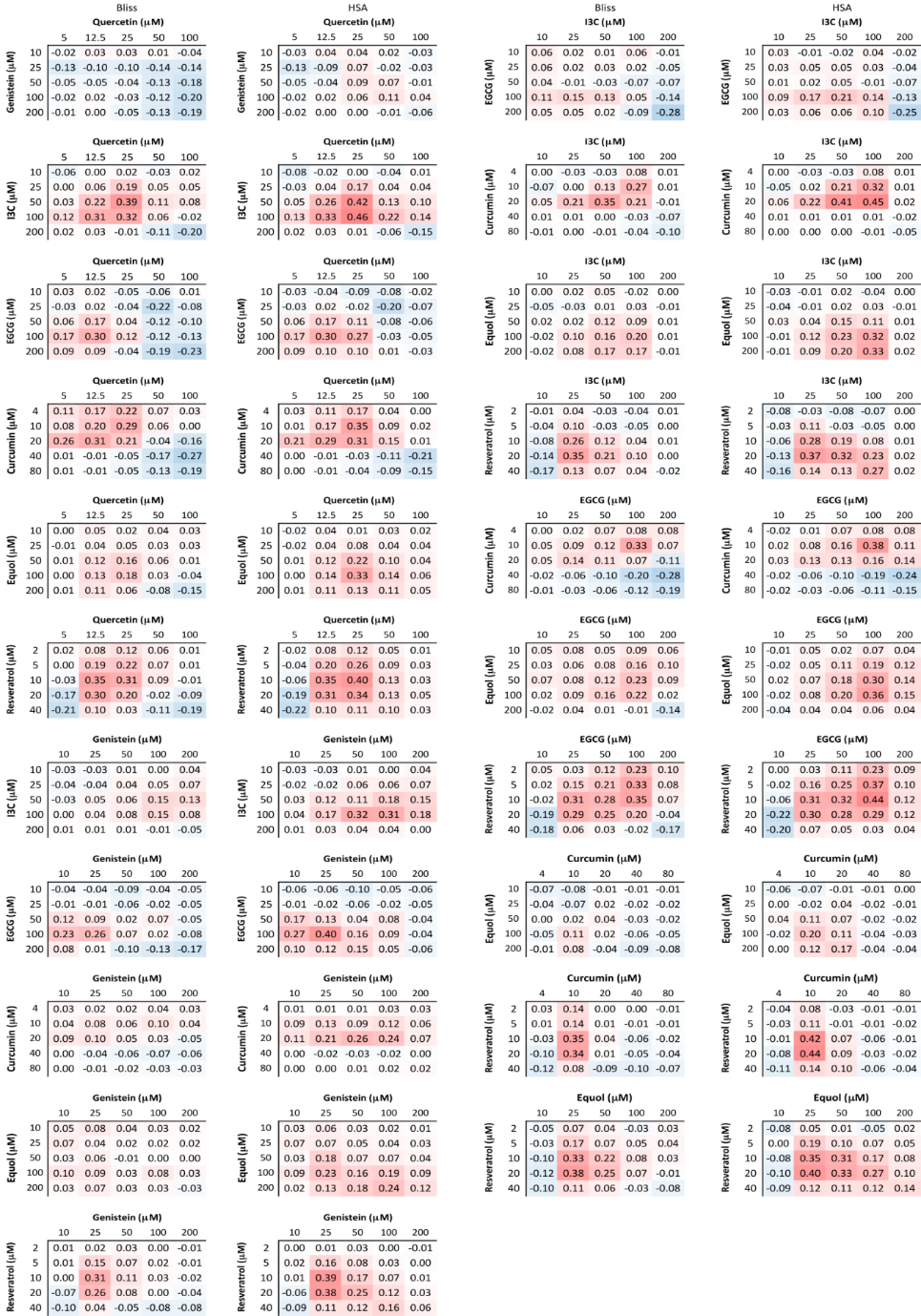


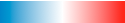


-1.0 0 1.0

Figure S3. Effect of combination treatment on prostate cancer cell viability. A. LNCaP, B. PC-3 or C. DU145 cells were treated with phytochemicals in combination, cell viability determined and data analysed for synergy or antagonism by either the Bliss or HSA model as described (Fig. S2).

**
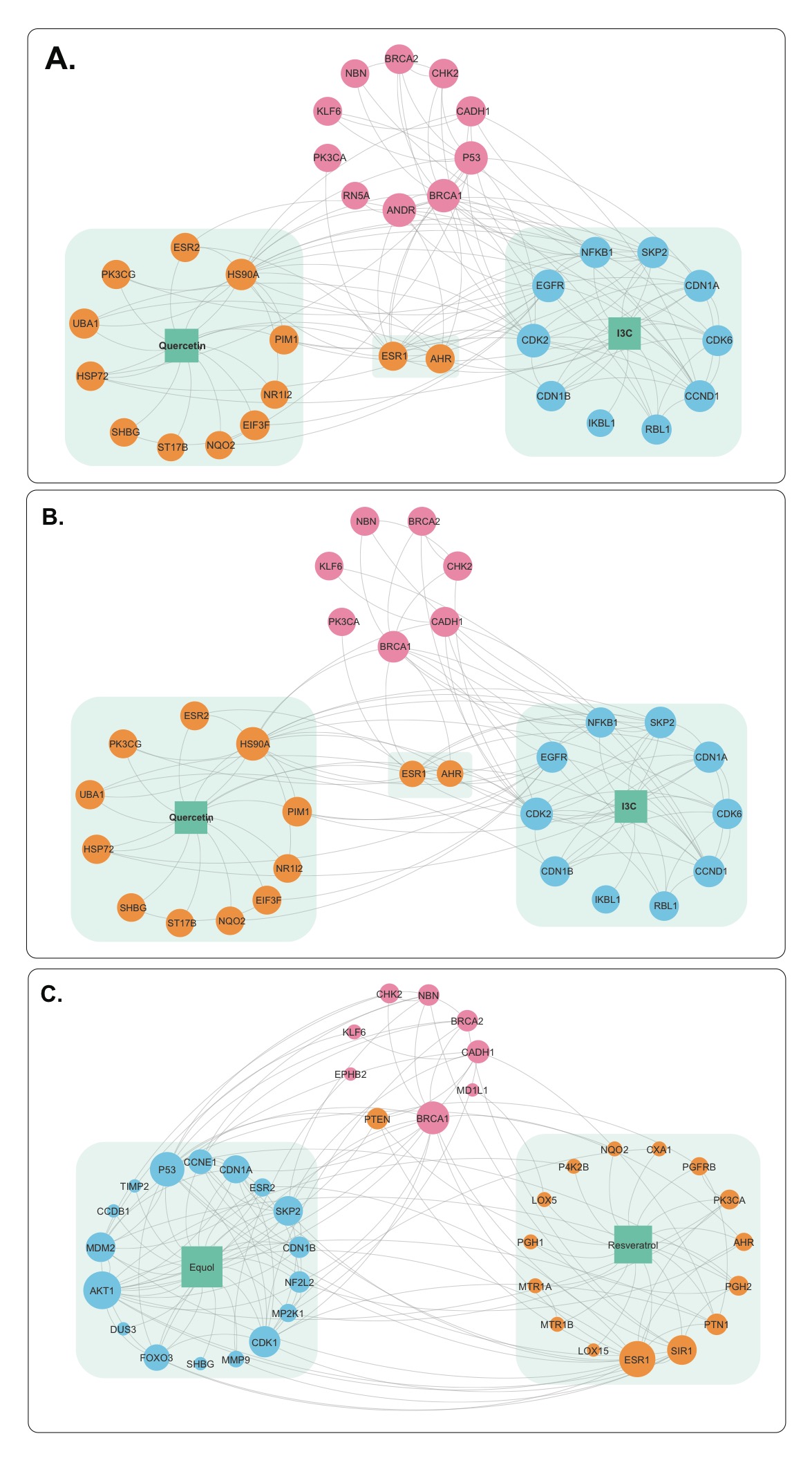
**

Figure S4. Phytochemical interaction network for the most potent synergistic combination in A. LNCaP cells, B. PC-3 cells and C. DU145 cells. Prostate cancer interactome has been adjusted for genotypic changes present in each cell line. LNCaP, Androgen receptor positive, pTEN negative, p53 positive; PC-3, androgen receptor negative, pTEN negative, p53 negative; DU145, androgen receptor negative, pTEN positive, p53 negative.
